# The joint influence of marital status, interpregnancy interval, and neighborhood on small for gestational age birth: a retrospective cohort study

**DOI:** 10.1186/1471-2393-8-7

**Published:** 2008-02-28

**Authors:** Nathalie Auger, Mark Daniel, Robert W Platt, Zhong-Cheng Luo, Yuquan Wu, Robert Choinière

**Affiliations:** 1 Unité Études et analyses de l'état de santé de la population, Institut national de santé publique du Québec, Montréal, Québec, Canada; 2 Département de médecine sociale et préventive, Université de Montréal, Montréal, Québec, Canada; 3 School of Health Sciences, University of South Australia, Adelaide, Australia; 4 Department of Epidemiology and Biostatistics, McGill University, Montréal, Québec, Canada; 5 Hospital Sainte Justine Research Centre, Department of Obstetrics and Gynaecology, Université de Montréal, Montréal, Québec, Canada

## Abstract

**Background:**

Interpregnancy interval (IPI), marital status, and neighborhood are independently associated with birth outcomes. The joint contribution of these exposures has not been evaluated. We tested for effect modification between IPI and marriage, controlling for neighborhood.

**Methods:**

We analyzed a cohort of 98,330 live births in Montréal, Canada from 1997–2001 to assess IPI and marital status in relation to small for gestational age (SGA) birth. Births were categorized as subsequent-born with *short *(<12 months), *intermediate *(12–35 months), or *long *(36+ months) IPI, or as firstborn. The data had a 2-level hierarchical structure, with births nested in 49 neighborhoods. We used multilevel logistic regression to obtain adjusted effect estimates.

**Results:**

Marital status modified the association between IPI and SGA birth. Being unmarried relative to married was associated with SGA birth for all IPI categories, particularly for subsequent births with *short *(odds ratio [OR] 1.60, 95% confidence interval [CI] 1.31–1.95) and *intermediate *(OR 1.48, 95% CI 1.26–1.74) IPIs. Subsequent births had a lower likelihood of SGA birth than firstborns. *Intermediate *IPIs were more protective for married (OR 0.50, 95% CI 0.47–0.54) than unmarried mothers (OR 0.65, 95% CI 0.56–0.76).

**Conclusion:**

Being unmarried increases the likelihood of SGA birth as the IPI shortens, and the protective effect of *intermediate *IPIs is reduced in unmarried mothers. Marital status should be considered in recommending particular IPIs as an intervention to improve birth outcomes.

## Background

The relationship between interpregnancy interval (IPI) and perinatal health is receiving increasing attention. A recent meta-analysis concluded that the IPI, defined as time between the last delivery and conception of the current pregnancy, can be associated with adverse birth outcomes when the IPI is either too short or too long [1]. The promotion of appropriate pregnancy spacing has been recommended to achieve better birth outcomes [2].

Although research on the IPI has been performed in many countries, relatively few studies have been conducted in developed nations characterized by low rates of adverse birth outcomes and comprehensive health insurance such as Canada [1]. Furthermore, studies on the IPI have not accounted for residential neighborhood which has been shown to independently predict birth outcomes [3-12]. Second, most studies have excluded firstborns from analyses of the IPI, thereby precluding the opportunity to explicitly compare risks across the full spectrum of birth orders. The need to consider a full spectrum of birth orders is important in evaluating effect modification by a third variable according to which the relationship between birth order and birth outcome may vary. The influence of IPI on small for gestational age (SGA) birth varies according to race [13], but moderation by other sociodemographic variables has not been assessed. Marital status is one influence increasingly recognized as a risk factor for adverse perinatal health outcomes [14-16] potentially operating though social support or stress mechanisms [17]. The influence of marital status on SGA birth, a birth outcome known to be associated with psychosocial factors such as social support [18,19], has yet to be fully understood. Being unmarried has been reported to increase the likelihood of SGA for subsequent-born relative to firstborn infants [17].

Given the above gaps in knowledge concerning the IPI, we sought to determine the relationship between marriage, firstborn birth and subsequent birth categorized according to IPI, and the likelihood of SGA birth, accounting for residential neighborhood cluster variations. We assessed whether effect modification was present between IPI category and marital status, adjusting for neighborhood. The setting was Montréal, a large Canadian city in which SGA birth has been shown to vary according to neighborhood [20].

## Methods

### Data

Data were drawn from the live birth registry for the province of Québec, Canada for the years 1997 to 2001. All births to mothers with a residential 6-digit postal code for the city of Montréal were extracted for analysis (n = 102,461). The Québec birth registry contains the date of birth of the index and previous birth, but not the conception date. The conception date was calculated by subtracting the gestational age (in weeks, based on ultrasound estimates) from the date of birth of the index child. The IPI was then calculated as the months between the conception date and the date of the previous birth. The IPI was used to group births into categories defined as: firstborn, subsequent-born with *short *IPI (less than 12 months), subsequent-born with *intermediate *IPI (12 to 35 months), and subsequent-born with *long *IPI (36 months or more). We did not evaluate very short IPIs because of sample size restrictions. Marital status was defined as being legally married versus not legally married. The following covariates were available for mothers: age (less than 20 years, 20 to 34.9 years, and 35 years and older), education (in continuous years, verified for log-linearity with SGA), country of birth (Canada-born versus foreign-born), and year of birth. The Québec birth registry does not include data on smoking or pregnancy complications. The outcome was defined as SGA birth (below the 10^th ^percentile using updated Canadian birth weight for gestational age and sex reference values) versus not SGA [21].

The complete Canadian 6-digit postal code was used to assign mothers to a police district, the administrative unit used to represent "neighborhood" [22]. Thus the data were arranged in a 2-level hierarchical structure with births (level-1) nested within 49 neighborhoods (level-2). We used Montreal police districts because they were created based on functionality, spatial homogeneity, and historic socio-demographic similarity of residents (average population = 37,000 residents/district) [23]. We accounted for two neighborhood characteristics associated with the birth outcome: 1) perception of security in the neighborhood, and 2) proportion immigrant population [23]. Police districts were grouped into quintiles from lowest to highest for both neighborhood variables.

### Exclusion criteria and missing data

Maternal education was missing for 7,347 births (7.2%). Deterministic imputation was used to replace these missing data using the mean maternal education of the specific postal code area within the police district. The imputation procedure left 23 births with missing maternal education; these values could not be imputed because of the absence of maternal education data in the given postal codes. Maternal country of birth was missing for 1,344 births (1.3%), and the postal code was invalid for 33 births. SGA status could not be determined for 124 births because of implausible gestational age [21]. There were 2,642 multiple births. We excluded multiple births and births with missing country of birth, postal code, SGA status, or maternal education values still missing after imputation, leaving a final sample of 98,330 singleton births.

### Statistical analysis

We used multi-level multivariate logistic regression models to estimate adjusted odds ratios, with births (individual-level) clustered within neighborhoods (specified as the random effect) [24]. Because the null 2-level model showed significant area-level variation (covariance parameter estimate = 0.0257, *p *< 0.0001), we retained this model. The area-level variation corresponded to an intra-class correlation of 0.8%, calculated according to the linear threshold model method [25]. The covariance parameter estimate corresponded to a median odds ratio of 1.16, indicating that, should a mother move to a neighborhood with a higher probability of SGA, her odds of having a SGA infant would in median increase 1.16 times. These odds for the influence of neighborhoods are comparable to those in other studies [25].

Individual-level variables were added to the model, followed by neighborhood-level variables. Last, we tested individual-level interactions between IPI category and marital status, and between these variables and other relevant variables. The significance of parameter estimates was assessed using the Wald test. We explored various cut- points for the IPI: 1–12, 13–30, 31+ months; 1–11, 12–23, 24+ months; 1–8, 9–30, 31+ months; and 1–8, 9–35, 36+ months. We chose the <12, 12–35, 36+ month cut-offs based on sufficient sample size in each category, as well as clinical relevance. In addition, we calculated population attributable fractions [26]. Analyses were conducted using SAS 9.0 (SAS Institute Inc, Cary/NC, 2002), with the GLIMMIX macro for multi-level logistic regression analyses [27].

This study was conducted through a mandate to monitor and research population health in the province of Québec, Canada, authorized by the Health Ministry and approved by the Québec Public Health Ethics Committee.

## Results

### Characteristics of mothers and infants

A large proportion of mothers were unmarried (39.8%, Table 1). The proportion of SGA to total births was higher in unmarried (10.1%) compared to married mothers (8.6%, Table 1). Slightly more than half (51.4%) of all newborns were subsequent births. Among married mothers, 56.8% of births were subsequent-born, whereas among unmarried mothers 43.2% were subsequent-born. For subsequent born infants, the IPI was *intermediate *for the majority (44.7%), followed by *long *(37%), and *short *(18.3%). Among subsequent births, SGA birth was least frequent when the IPI was *intermediate *(6.6%) compared to *short *(7.4%) or *long *(8.2%, Table 2). Married mothers had more subsequent births with an *intermediate *IPI (26.6%) compared to unmarried mothers (17.4%, Table 1).

**Table 1:** Characteristics of mothers, infants, and neighborhoods according to marital status, singleton births, Montréal, Canada, 1997 to 2001

**Characteristic**	**Married**		**Unmarried**		**Total births**	
	
	**n**	**%**	**n**	**%**	**n**	**%**
**Mothers**						
Age						
<20 years	582	1.0	3388	8.6	3970	4.0
20–34 years	46606	78.8	29836	76.2	76442	77.7
35+ years	11962	20.2	5956	15.2	17918	18.2
Education (years)	59150	14 (4)*	39180	13 (3)*	98330	14 (4)*
Maternal place of birth						
Canadian-born	24963	42.2	29985	76.5	54948	55.9
Foreign-born	34187	57.8	9195	23.5	43382	44.1
**Infants**						
IPI category						
Firstborn	25564	43.2	22265	56.8	47829	48.6
Subsequent born						
Short IPI	6045	10.2	3190	8.1	9235	9.4
Intermediate IPI	15760	26.6	6808	17.4	22568	23.0
Long IPI	11781	19.9	6917	17.7	18698	19.0
Growth						
Normal growth	54087	91.4	35219	89.9	89306	90.8
SGA	5063	8.6	3961	10.1	9024	9.2
**Neighborhoods**						
Perceived security						
High	13715	23.2	6071	15.5	19786	20.1
High-moderate	14074	23.8	6653	17.0	20727	21.1
Moderate	12813	21.7	9849	25.1	22662	23.1
Low-moderate	10265	17.4	7959	20.3	18224	18.5
Low	8283	14.0	8648	22.1	16931	17.2
Proportion foreign-born						
High	16015	27.1	5135	13.1	21150	21.5
High-moderate	13935	23.6	4831	12.3	18766	19.1
Moderate	11495	19.4	8163	20.8	19658	20.0
Low-moderate	11083	18.7	8532	21.8	19615	20.0
Low	6622	11.2	12519	32.0	19141	19.5
Total live births	59150	60.2	39180	39.8	98330	100

* Values represent the mean (standard deviation).

**Table 2:** Characteristics of mothers, infants, and neighborhoods according to SGA status, singleton births, Montréal, Canada, 1997 to 2001

**Characteristic**	**Normal growth**		**SGA**	
	
	**n**	**%**	**n**	**%**
**Mothers**				
Age				
<20 years	3484	87.8	486	12.2
20–34 years	69497	90.9	6945	9.1
35+ years	16324	91.1	1593	8.9
Education (years)	89306	14 (4)*	9024	13 (4)*
Maternal place of birth				
Canadian-born	50209	91.4	4739	8.6
Foreign-born	39097	90.1	4285	9.9
Marital status				
Married	54087	91.4	5063	8.6
Unmarried	35219	89.9	3961	10.1
**Infants**				
IPI category				
Firstborn	42508	88.9	5321	11.1
Subsequent born				
Short IPI	8555	92.6	680	7.4
Intermediate IPI	21081	93.4	1487	6.6
Long IPI	17162	91.8	1536	8.2
**Neighborhoods**				
Perceived security				
High	18241	92.2	1545	7.8
High-moderate	18905	91.2	1822	8.8
Moderate	20514	90.5	2148	9.5
Low-moderate	16585	91.0	1639	9.0
Low	15061	89.0	1870	11.0
Proportion foreign-born				
High	18931	89.5	2219	10.5
High-moderate	17200	91.7	1566	8.3
Moderate	17935	91.2	1723	8.8
Low-moderate	17981	91.7	1634	8.3
Low	17259	90.2	1882	9.8
Total live births	89306	90.8	9024	9.2

* Values represent the mean (standard deviation).

### Characteristics of neighborhoods

There was an inverse relationship between unmarried status and neighborhood perceived security (Table 1), and this coincided with an increasing frequency of SGA birth as perceived security diminished in neighborhoods (Table 2). Table 3 shows that the IPI for subsequent births varied according to neighborhood characteristics. High neighborhood perceived security corresponded to more frequent *intermediate *IPI (49.5%) and less frequent *short *(16.7%) or *long *(33.8%) IPIs, relative to neighborhoods with low perceived security (41.0%, 19.7%, and 39.3%, respectively).

**Table 3:** Interpregnancy interval for subsequent births according to neighborhood characteristics, singleton births, Montréal, Canada, 1997–2001

	**Total subsequent births**	**Short IPI**		**Intermediate IPI**		**Long IPI**	
	
	**n**	**n**	**%**	**n**	**%**	**n**	**%**
**Perceived security**							
High	10838	1807	16.7	5366	49.5	3665	33.8
High-moderate	10878	2014	18.5	4996	45.9	3868	35.6
Moderate	11348	2026	17.9	4886	43.1	4436	39.1
Low-moderate	9242	1772	19.2	3958	42.8	3512	38.0
Low	8195	1616	19.7	3362	41.0	3217	39.3
**Proportion foreign-born**							
High	11611	2260	19.5	4890	42.1	4461	38.4
High-moderate	9973	1687	16.9	4580	45.9	3706	37.2
Moderate	10172	1946	19.1	4486	44.1	3740	36.8
Low-moderate	9971	1759	17.6	4779	47.9	3433	34.4
Low	8774	1583	18.0	3833	43.7	3358	38.3

### Multi-level analysis

**Figure 1:**
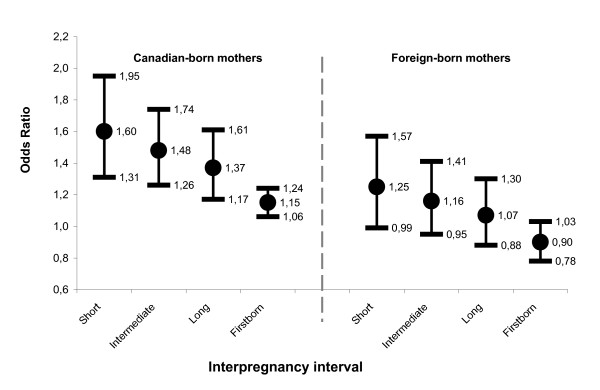
**Odds of SGA birth for unmarried relative to married mothers, according to interpregnancy interval and maternal place of birth, singleton births, Montréal, Canada, 1997 to 2001**. Results are from multi-level logistic regression testing an interaction term between marital status and interpregnancy interval, adjusting for maternal age, education, country of birth, year of birth, interaction between marital status and country of birth, neighborhood perceived security, and neighborhood proportion foreign-born population. Odds ratios are for unmarried relative to married mothers.

**Figure 2:**
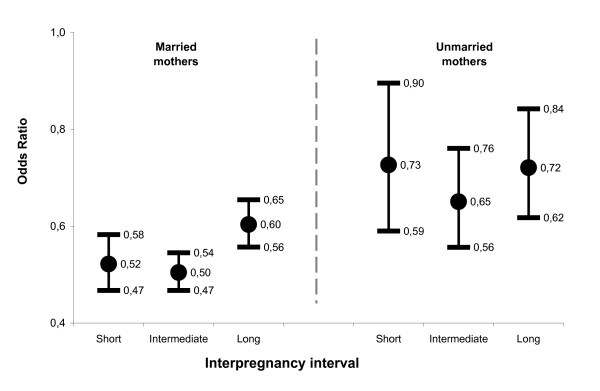
**Odds of SGA birth for interpregnancy interval relative to firstborns, stratified by marital status, singleton births, Montréal, Canada, 1997 to 2001**. Results are from multi-level logistic regression testing an interaction term between marital status and interpregnancy interval, adjusting for maternal age, education, country of birth, year of birth, interaction between marital status and country of birth, neighborhood perceived security, and neighborhood proportion foreign-born population. Odds ratios are for interpregnancy interval relative firstborns.

Marital status and IPI category were both independently associated with SGA birth. In addition, marital status modified the influence of IPI category on SGA birth. Effect modification was also present between marital status and maternal country of birth. Effect modification was not present between individual and neighborhood variables. Figures 1 and 2 display odds ratios for levels of main effects adjusted for maternal age, education, country of birth, infant year of birth, perception of neighborhood security, and proportion immigrant population.

#### Being unmarried

For Canadian-born mothers, unmarried relative to married mothers had significantly greater odds of having a SGA birth in all IPI categories (*i.e*., confidence intervals exclude one, Figure 1). Being unmarried was a stronger risk factor among subsequent births (pooled odds ratio [OR] 1.47, 95% confidence interval [CI] 1.28–1.68, not shown in figure) than among firstborns (OR = 1.15, 95% CI 1.06–1.24). Furthermore, odds associated with being unmarried were greater among subsequent births with both *short *(OR = 1.60, 95% CI 1.31–1.95) and *intermediate *IPIs (OR = 1.48, 95% CI 1.26–1.74) than among firstborns (Figure 1). For subsequent births, the magnitude of effects associated with being unmarried decreased with longer IPIs.

For foreign-born mothers, unmarried relative to married mothers did not have a significantly higher likelihood of SGA birth (*i.e*., confidence intervals do not exclude one). Nevertheless, among foreign-born mothers, a similar pattern of decreasing odds of SGA birth associated with marital status was observed with increasing IPI (Figure 1).

#### Being a subsequent birth

Figure 2 illustrates the protective effects of being a subsequent birth compared to being firstborn. For both married and unmarried mothers, subsequent births had a lower likelihood of SGA birth, irrespective of the IPI. Furthermore, the lesser odds of SGA for subsequent births were more substantial among married women compared to unmarried women. These differences were statistically significant (*i.e*., confidence intervals do not overlap) for subsequent births with *intermediate *(OR_married _= 0.50, 95% CI 0.47–0.54 versus OR_unmarried _= 0.65, 95% CI 0.56–0.76) and *short *IPIs (OR_married _= 0.52, 95% CI 0.47–0.58 versus OR_unmarried _= 0.73, 95% CI 0.59–0.90).

#### Having long or short IPIs

For married mothers, subsequent births with *intermediate *IPIs were more protective (OR = 0.50, 95% CI 0.47–0.54) than those with *long *IPIs (OR = 0.60, 95% CI 0.56–0.65). The likelihood of a SGA birth for subsequent births with *short *IPIs was not significantly different from those with *intermediate *IPIs. For unmarried mothers, there was no difference between the likelihood of a SGA birth among the three subsequent birth IPI categories, although the *intermediate *IPI corresponded to the lowest odds of SGA birth (Figure 2).

### Population attributable fractions

The fraction of SGA birth related to being unmarried was 5.3%. In contrast, the fraction of SGA birth related to a short or long IPI was of lesser magnitude (3.2%). Firstborns accounted for 26% of the population risk.

## Discussion

Our study confirms the results of other studies that have found marital status and IPI to be associated with adverse birth outcomes [1,2,14-16,28-31], and provides additional insights on factors contributing to SGA birth. First, by including neighborhood factors as explanatory variables, we were able to demonstrate that the influence of the IPI on SGA birth varies according to features of the neighborhood. This finding is consistent with the growing literature on neighborhoods and health [3-12,32,33].

Second, through testing for effect modification between individual predictor variables, we demonstrated that the association between IPI and SGA birth depends on maternal marital status. We also showed that the association between marital status and SGA birth varied according to IPI and maternal place of origin (Canadian-born versus foreign-born). Specifically, we found that the likelihood of SGA birth associated with being unmarried was highest for subsequent births compared to firstborns, especially for *short *IPIs. This association was strongest for Canadian-born mothers. Foreign-born mothers might be less susceptible to health-related consequences associated with being unmarried. We are aware of two previous studies reporting that being unmarried is a greater risk factor for adverse birth outcome in subsequent births compared to firstborns; however, these studies did not address the IPI [17,28].

Another key finding was that the odds of SGA birth conferred by being unmarried tended to be similar to that of firstborns as the IPI increased. Different mechanisms may be involved. Perhaps the presence of young siblings (*i.e*., *short *IPI) in a household contributes extra stress to unmarried mothers, thereby negatively impacting the pregnancy environment. In the case of large age gaps between siblings (*i.e*., *long *IPI), it might be that that child rearing stresses are diminished and resemble those of unmarried mothers without children. Such a mechanism suggests that a marital partner may be important for diminishing stress associated with caring for younger children. The exact nature of such stressors (*e.g*., fewer stressors, better coping or adaptation) remains to be elucidated, however. Also, other unmeasured socio-economic status indicators may partly explain, or confound the observed associations. Maternal age cannot explain the associations because we adjusted for this variable. An alternative interpretation for the influence of marital status is that nutritional depletion may be present in mothers with *short *IPIs [34]; such mothers may be more susceptible to any effects of being unmarried. Mothers with *long *IPIs may have had sufficient time to restore nutritional reserves, which may in turn help buffer any adverse effects of being unmarried. Other biological mechanisms may also link the psychosocial stress of being unmarried with the likelihood of SGA birth [18,19], and may operate through neuroendocrine or immune pathways known to be influenced by psychological stress [35,36].

Our study confirmed that firstborns are at greater risk of being SGA than their siblings [37]. Furthermore, our data indicate that the protective effects of being a subsequent birth are greater for infants born to married compared to unmarried women. Being married appears to augment the protective effects of a multiparous uterine environment. This finding is difficult to explain, and we suspect that marriage may serve as a proxy for other determinants of SGA birth. It is well known that unmarried mothers are more likely to have unfavorable lifestyles (*e.g*., smoking) associated with lower socioeconomic status. These and other unmeasured risk factors linked to being unmarried may account for or partly mediate the lesser protective effects of IPI among unmarried women.

Lastly, our study confirms the association between IPI and SGA birth [1,38,39]. Our novel finding is that this association varies depending on marital status. More specifically, *intermediate *IPIs were significantly more protective than *long *IPIs for married mothers only. Thus our results support the recommendation that mothers should avoid prolonged IPIs, but this applies, for unknown reasons, primarily to married mothers. Our data do not support the finding that *short *IPIs are associated with a greater risk of SGA, and this applies for both married as well as unmarried mothers. We did not evaluate *extremely short *IPIs in this study.

Beyond the influence of IPI on SGA birth, marital status is an especially strong predictor of this outcome. While we suggest neither a causal association nor a strict interpretation of "attributable risk", the estimated attributable fractions indicate that being unmarried (population attributable fraction = 5.3%) is a more important contributor to SGA birth than *short *or *long *IPIs (population attributable fraction = 3.2%). One study reported an attributable risk of 9.4% for *short *or *long *IPIs, but because the study was restricted to subsequent-born infants (*i.e*. excluded firstborns) and did not consider marriage, this estimated attributable risk cannot be directly compared with ours [34].

Our study may be subject to several limitations. First, we used broad categorizations of marital status and IPI which may inadvertently mask underlying associations. For example, because our data did not permit finer categorization, we defined "unmarried" as not having a legal marital arrangement; however sub-groups of unmarried women such as those in stable cohabitation may be subject to different associations. Similarly, we categorized foreign-born mothers as one group when in fact differences may exist based on nationality or length of residence in Canada, but this was unavoidable because data on duration of residence is not available in the birth registry. Second, we used an administrative definition of neighborhood that may not correspond to residents' perception of neighborhood; effect estimates might differ for other neighborhood boundaries. Third, we do not have data on potential confounders such as infertility treatment which may partly account for the observed associations, although we excluded multiple births [40]. We do not know how factors such as income or alternate classifications of socio-economic status could influence our results. We also could not correct reduced precision resulting from correlation between siblings as our data do not allow the identification of siblings, although we do not suspect this effect could be substantial. Last, the extent to which our results might be generalizable to other populations is unknown. Nevertheless, these limitations are countered by a large sample size, representing all births over five years in a large Canadian city.

## Conclusion

Our results have a number of implications for current infant health promotion practices. Present obstetric guidelines focus on promoting an appropriate IPI (*i.e*., *intermediate *IPI) to mothers contemplating subsequent pregnancies. Our results suggest that married mothers may be more likely to benefit from such recommendations than unmarried mothers. Thus, prevention strategies for unmarried mothers may well need to differ from those for married mothers.

Differential benefits to married mothers may be compounded when we consider that the IPI is associated with the social characteristics of the neighborhood (Table 3). Although no other study has yet addressed neighborhood influences on the association between IPI and birth outcomes, many studies have found neighborhoods to be important for perinatal health outcomes [3-12]. Thus, prevention strategies may need to take neighborhood factors into account.

Our results bring into question current public health recommendations in obstetrics that appropriate IPIs should be emphasized as an important intervention for newborn health for all women. Focusing on the IPI as an intervention may be differentially successful depending on the social group a mother belongs to. In fact, unmarried mothers who are most at risk of SGA birth may be the least likely to benefit from such an intervention. Marital status in particular might need to be accounted for in prevention strategies for improving birth outcomes.

## Abbreviations

CI: Confidence interval; IPI: Interpregnancy interval; OR: Odds ratio; SGA: Small-for-gestational-age.

## Competing interests

The authors declare that they have no competing interests.

## Authors' contributions

NA developed the research design, guided the data analysis, interpreted the results, and wrote the manuscript. MD, RWP, and ZCL contributed to the research design, analysis and review of the manuscript. YW performed the statistical analysis. RC contributed to conception pf the study and development of the research design. All authors have seen and approved the final version of the manuscript.

## Pre-publication history

The pre-publication history for this paper can be accessed here:


